# Maxillofacial Fractures: A Four-Year Retrospective Study of 1828 Cases in West China

**DOI:** 10.7759/cureus.40482

**Published:** 2023-06-15

**Authors:** Rong Miao, Jiankang Zhang, Jing Zhou, Xiaoning Qiu, Gang Liu, Xinzhi Tan, Junming Tao, Baohua Yang, Lei Liu, Wei Tang, Jie Long, Wei Jing

**Affiliations:** 1 Oral and Maxillofacial Surgery, State Key Laboratory of Oral Diseases & National Clinical Research Center for Oral Diseases & Department of Oral and Maxillofacial Surgery, West China Hospital of Stomatology, Sichuan University, Chengdu, CHN; 2 Oral and Maxillofacial Surgery, The Affiliated Stomatology Hospital of Southwest Medical University, Luzhou, CHN

**Keywords:** retrospective study, large series report, trauma, epidemiology, maxillofacial fracture

## Abstract

Objective: To analyze the epidemiological and clinical characteristics of maxillofacial fracture cases in a stomatological center in southwest China.

Methods: This study includes 1828 cases of maxillofacial fractures treated in our hospital from January 2018 to December 2021. We analyzed the gender, age, causes of injury, fracture sites, concomitant injuries, treatment, and postoperative infection of these cases. Our data are also compared with those from similar domestic studies.

Results: Among the 1828 cases, the male-to-female ratio was 2.48:1 with an average age of 34.55 ± 16.36 years. The highest incidence of fracture was 21-50 years old, and the most common cause of injury was falls (38.95%). There was a statistically significant difference in the composition of injury causes among different age groups(P<0.05). Mandible (37.56%) was the most easily fractured site, and limb injury (17.89%) was the most common concomitant body injury. In all cases, 85.23% of patients were treated with open reduction and internal fixation.

Conclusions: Maxillofacial fractures often occur in the mandible of young and middle-aged men. Falls and traffic accidents are the main causes of injury, often accompanied by limb and brain injuries. Open reduction and internal fixation is still the most commonly used treatment. There are some differences in the results reported by different domestic hospitals.

## Introduction

Maxillofacial fractures are prone to cause facial deformities and stomatognathic dysfunctions that affect patients' social activities and quality of life, further leading to economic difficulties and psychological problems [1-3]. Craniocerebral injury, dyspnea, massive hemorrhage, eye trauma, and cervical vertebral fractures may also occur, and the patient's life may even be threatened in severe cases because of the special anatomical location of the jawbone [4-8]. For children in the peak stage of growth, fractures involving the growth and development center of the jawbone may also have irreversible effects on facial growth and development [9]. Therefore, it is necessary to inductively analyze the characteristics of maxillofacial fractures to provide a reference for their clinical diagnosis, treatment, and prevention.

Because of differences in social environments and other factors, maxillofacial fractures may show regional differences in terms of their causes, epidemiological characteristics, and clinical treatment methods [10,11]. Large series reports of maxillofacial fractures from different regions are of great value for further analysis of the clinical characteristics of such fractures and for optimizing the means of disease prevention and control [12,13]. In the present study, we retrospectively analyzed 1828 patients with maxillofacial fractures hospitalized at West China Hospital of Stomatology, Sichuan University, from January 2018 to December 2021 and summarized the clinical characteristics and overall treatment methods of these fractures in our center. Our goal in this study was to provide a reference for the in-depth study of maxillofacial fractures.

## Materials and methods

Materials

The data of patients with maxillofacial fractures hospitalized in the Department of Oral and Maxillofacial Surgery of West China Hospital of Stomatology, Sichuan University, from January 2018 to December 2021, were collected and analyzed. The West China Hospital of Stomatology Institutional Review Board (WCSHIRB) approved the study protocol.

The inclusion and exclusion criteria were as follows: The patients included in this study were required to have complete records, specialist examination details, and complete surgical records for those who underwent surgery. Simple soft tissue trauma and patients with maxillofacial fractures admitted to the hospital to repair posttraumatic deformities will be excluded from the experiment.

Methods

Data is collected by consulting our hospital's electronic medical records. The statistical content includes the patient's name, medical record number, gender, age, course of the disease, cause of injury, fracture sites, comorbidities, treatment methods, and postoperative infections. The name and medical record number are for retrieval purposes only, and the specific grouping of other data is as follows: (1) Age: 0-10 years old; 11-20 years old; 21-30 years old; 31-40 years old; 41-50 years old; 51-60 years old; 61 years old and above; (2) Causes of injury: falls; traffic accidents; falls from a height (including falling from tall buildings and other high places such as mountains or trees); assaults (comprising of interpersonal violence, some sporting accidents, and some industrial accidents); incision and puncture wounds; animal attacks; firearm explosions; miscellaneous causes; (3) fracture sites: mandible; maxilla and hard palate; zygoma and zygomatic arch; orbital; nasal; (4) Systemic concurrent injury: limb injury; craniocerebral injury; chest and back injury; neck injury; spine injury; eye injury; abdominal injury; pelvic injury.

And the diagnostic criteria for postoperative infection are as follows: (1) no preoperative infection; (2) formation of pus, fistula, or fluctuation in the maxillofacial region; (3) physical examination combined with laboratory examination results support the diagnosis of postoperative infection.

Statistical analysis

Statistical analysis was performed using SPSS 21.0 (IBM Corp., Armonk, NY). Different statistical analysis methods are used for different types of data. Mean, and standard deviation were calculated for quantitative variables like age, while frequency and percentage were calculated for qualitative variables like gender, causes of injury, site of fractures, and systemic concurrent injury. The chi-square test was used to analyze the association between age, gender, and causes of injury. A P-value <0.05 was considered statistically significant.

## Results

General information

Of the 1828 patients with maxillofacial fractures included in this study, the number of cases per year from 2018 to 2021 was 487, 503, 374, and 464, respectively. In 2020, the number of cases was less than that in the other three years because of the impact of the COVID-19 pandemic. The study population comprised 1302 (71.23%) male patients and 526 (28.77%) female patients, with a male: female ratio of 2.48:1.00. One sample proportion z-test with the null hypothesis of an equal proportion of males and females was performed in all patients and the age groups (Table 1).

**Table 1:** Gender distribution of 1828 cases of maxillofacial fractures a: P-value of one sample proportion z-test with the null hypothesis of an equal proportion of males and females.

	Male N (%)	Female N (%)	Group Percentage (%)	Male: Female radio	P-Value^a^
Total	1302 (71.23%)	526 (28.77%)	1	2.48:1.00	<0.001
0-10 years	80 (61.54%)	50 (38.46%)	130 (7.11%)	1.60:1.00	0.008
11-20 years	159 (66.25%)	81 (33.75%)	240 (13.13%)	1.96:1.00	<0.001
21-30 years	337 (76.24%)	105 (23.76%)	442 (24.18%)	3.21:1.00	<0.001
31-40 years	220 (72.13%)	85 (27.87%)	305 (16.68%)	2.59:1.00	<0.001
41-50 years	275 (72.75%)	103 (27.25%)	378 (20.68%)	2.67:1.00	<0.001
51-60 years	159 (70.35%)	67 (29.65%)	226 (12.36%)	2.37:1.00	<0.001
>60 years	72 (67.29%)	35 (32.71%)	107 (5.85%)	2.06:1.00	<0.001

**Figure 1:**
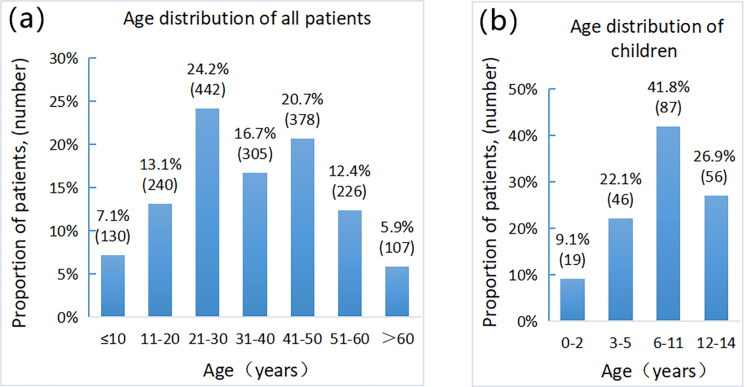
Age distributions. (a) The age distribution of all 1828 patients with maxillofacial fractures. The patients were analyzed in 10-year age groups, and the patients aged >60 years were considered one group. (b) The age distribution of pediatric patients. The children were divided into four age groups: 0 to 2, 3 to 5, 6 to 11, and 12 to 14. Of all four groups, the 6 to 11-year-old group had the highest proportion of children (41.8%). When this group was further divided into the 6 to 8-year-old group and the 9 to 11-year-old group, the group with the highest proportion of children was the 12 to 14-year-old group.

In this study, 1828 patients with maxillofacial fractures ranged from 10 months to 85 years, with a mean age of 34.55 ± 16.36 years. The age composition ratios were analyzed according to 10-year age groups, patients aged 21 to 30-year group (24.18%) and patients aged 41 to 50-year-old group (20.68%) were the highest proportions, whereas patients aged < 10-year-old (7.11%) and patients aged > 60-year-old group (5.85%) were the lowest proportions. More than half of the patients are aged 21 to 50 (61.54%). In different age groups, there was a statistically significant difference in gender distribution (P < 0.05). Figure 1a shows the number of patients in each age group.

Children were analyzed separately because the age groups of children should not be defined on a 10-year basis. Children were defined as patients aged ≤14 years and grouped with reference to school-age stages. The results showed that most children were 6 to 11 years old (87 patients, 41.83%), followed by 12 to 14 years old (56 patients, 26.92%), 3 to 5 years old (46 patients, 22.16%), and 0 to 2 years old (19 patient, 9.13%). Considering that the 6- to 11-year-old group had a larger age span than the other groups, it was unfair to compare the number of patients. When this group was further divided into two groups, namely the 6 to 8-year-old group and the 9 to 11-year-old group, then the former group had 50 (24.0%) patients, and the latter had 37 (17.8%) patients. The group with the highest number of children thus became the 12 to 14-year-old group (see Figure 1b).

Analysis of causes of injury

**Figure 2:**
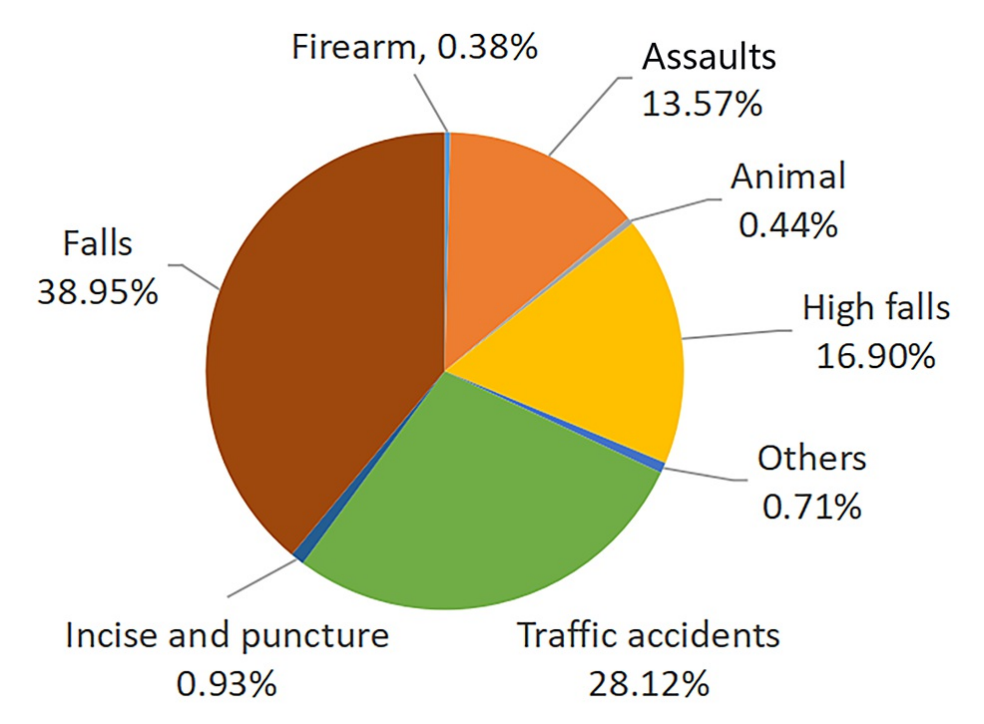
Etiology distribution of 1828 patients with maxillofacial fractures. The top four causes of injury were falls, traffic accidents, falls from a height, and assaults. (The pie chart is not arranged in sequence of data values because the four items with an incidence of <1% were too close to be clearly presented.)

In this study, the top three causes of injury were falls (712 patients, 38.95%), traffic accidents (514 patients, 28.12%), and falls from a height (319 patients, 16.90%). Other causes of injury were assaults (248 patients, 13.57%), incision and puncture wounds (17 patients, 0.93%), animal attacks (8 patients, 0.44%), firearm explosions (7 patients, 0.38%), and miscellaneous causes (13 patients, 0.71%), including pathological fractures, fractures caused by extraction of the third mandibular molar, and accidental bruises respectively, as shown in Figure 2. However, for the entire population, there were statistically significant differences in gender distribution among different etiological groups (P<0.05).

**Figure 3:**
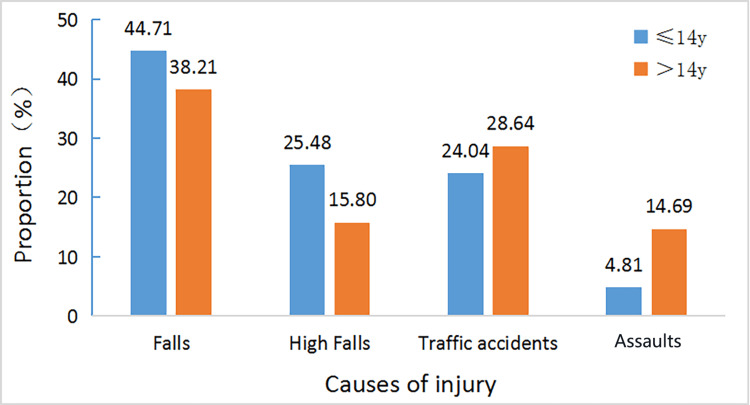
Comparison of the main causes of injury in patients aged ≤14 and >14 years

Among children, the cause of injury with the highest incidence was falls (93 patients, 44.71%), and this incidence was higher than that in the group of patients aged >14 years (38.21%). The cause of injury with the second highest incidence was falls from a height (53 patients, 25.48%), and this incidence was also higher than that in the group of patients aged >14 years (15.80%). The third most common cause was traffic accidents (50 patients, 24.04%), the incidence of which was lower than that in the group of patients aged >14 years (28.64%). The incidence of assaults resulting in maxillofacial fractures in children (4.81%) was also lower than that in the group of patients aged >14 years. There was a statistically significant difference in the composition of causes of injury among different age groups (P<0.05). The main causes of injury are shown in Figure 3.

Distribution of maxillofacial fracture sites

**Figure 4:**
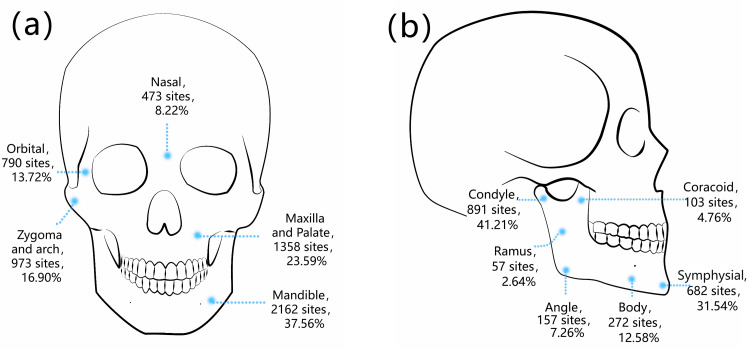
Distribution of maxillofacial fracture sites. (a) General distribution of the fracture sites. (b) The mandible was analyzed separately, and the proportions of fractures occurring in each part are presented.

Among the 1828 patients included in this study, 5756 fractures occurred, with an average of 3.15 fractures per patient. A single fracture was present in 410 (22.43%) patients, and multiple fractures were present in 1418 (77.57%). The largest number of fractures (2162, 37.56%) occurred in the mandible, 1358 (23.59%) occurred in the maxilla and hard palate, 973 (16.90%) occurred in the zygoma and zygomatic arch, 790 (13.72%) occurred at orbital sites, and 473 (8.22%) occurred at nasal sites (Fig. 4a).

To further investigate the distribution of different types of maxillofacial fractures, we used "A" to represent the maxilla and hard palate, "B" to represent the mandibular, "C" to represent the zygomatic orbital complex, and "D" to represent the nasal bone for further statistical analysis. The results showed that among the 1828 cases of maxillofacial fractures, the highest incidence was observed in patients with mandibular fractures only, accounting for 43.93%, followed by patients with fracture type AC, accounting for 20.59%, the third was patients with fracture type BC, accounting for 9.52%, the fourth was patients with fracture type ACD, accounting for 7.11%, and other types of fractures accounted for less than 6% each.

Given that the incidence of mandibular fractures was much higher than that at other sites, we conducted a separate analysis for mandibular fractures. Of all 2162 mandibular fractures, 682 (31.54%) were symphysial fractures, 891 (41.21%) were condylar fractures, 272 (12.58%) were mandibular body fractures, 157 (7.26%) was a mandibular angle fracture, 103 (4.76%) were coracoid fractures, and 57 (2.64%) were mandibular ramus fractures (Fig. 4b).

Considering the particularity of mandibular fractures, we analyzed separately the cases of mandibular fractures. For analysis, we assigned the letters "a" to the condyle, "b" to the angle, ramus, and coronoid of the mandible, and "c" to the symphysial and body of the mandibular. The findings revealed that among all cases of mandibular fractures, the "ac" type multiple fractures had the highest incidence (35.27%), followed by single fracture occurring in "c" (19.00%), "bc" type multiple fractures (14.51%), fractures occurring only in "a" (13.37%), single fracture occurring in "b" (8.80%), "abc" type multiple fractures (7.39%), and the least frequent being "ab" type multiple fractures (1.67%).

**Figure 5:**
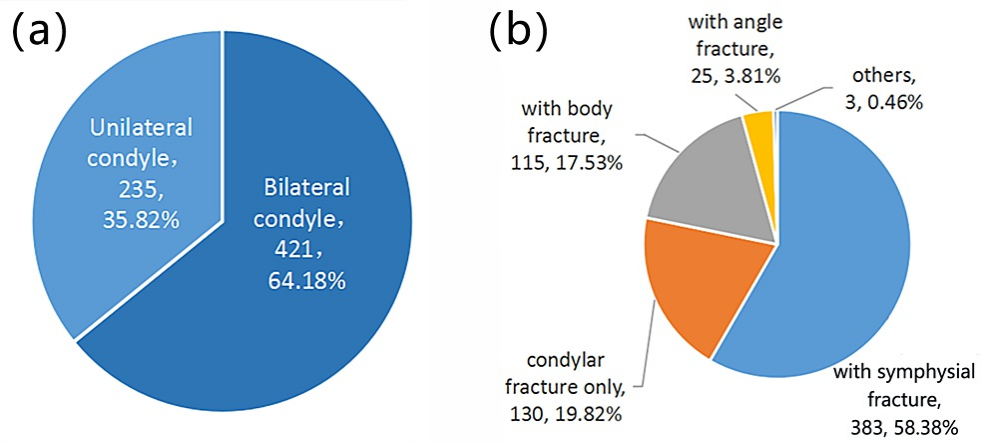
Occurrence of condylar fractures. (a) Number and proportion of unilateral and bilateral condylar fractures. (b) Condylar fractures with concomitant fractures of other parts of the mandible.

Among mandibular fractures, the treatment of condylar fractures is the most controversial [14]. Therefore, we further analyzed the occurrence of condylar fractures to provide a reference for their clinical diagnosis and treatment. Of the 656 patients with condylar fractures, there were 421 unilateral condylar fractures and 235 bilateral condylar fractures. Among the various parts of the mandible, there was 130 simple condylar fracture, 383 concomitant symphysial fractures, 25 concomitant mandibular angle fractures, and 115 concomitant mandibular body fracture (Fig 5).

For the 208 pediatric patients included in this study, 560 fractures occurred, with an average of 2.69 fractures per patient. A single fracture was present in 50 (24.04%) patients, and multiple fractures were present in 158 (75.96%). The largest number of fractures (383, 68.39%) occurred in the mandible, 75 (13.39%) occurred in the maxilla and hard palate, 44 (7.86%) occurred in the zygoma and zygomatic arch, 33 (5.89%) occurred at orbital sites, and 25 (4.46%) occurred at nasal sites.

For the 1620 patients over 14 years old in this study, 5196 maxillofacial fractures occurred, with an average of 3.21 fractures per patient. A single fracture was present in 360 (22.22%) patients, and multiple fractures were present in 1260 (77.78%). The largest number of fractures (1779, 34.24%) occurred in the mandible, followed by the maxilla and hard palate (1283,24.69%), the zygoma and zygomatic arch (929,17.88%), the orbital sites (757,14.57%), and the nasal sites (448, 8.62%).

Treatment method analysis

Of all 1828 patients with maxillofacial fractures, 839 (45.90%) patients underwent open reduction and internal fixation (ORIF) for each fracture, 719 (39.33%) patients underwent ORIF for a subset of fractures, and 270 (14.77%) patients underwent conservative treatments such as intermaxillary traction or observation. The patients who received conservative treatment mostly had been injured for more than three weeks, patients with high condylar fractures, and pediatric patients. In terms of ORIF materials, most patients were treated with titanium alloy bone plates, and a small number of patients (10.72%) were treated with absorbable bone plates. Notably, absorbable plates are only used for fractures in which the stress is small, or the displacement is not obvious.

Analysis of patients with concomitant injuries in other parts of the body

**Figure 6:**
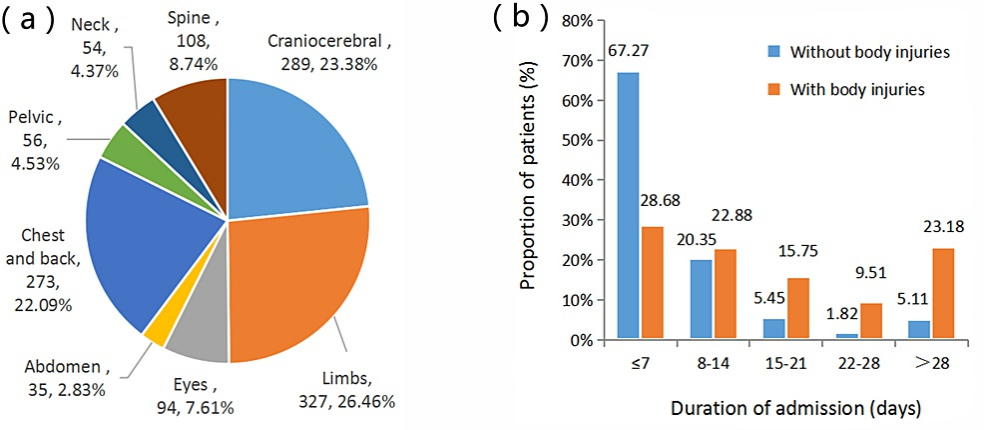
Concomitant injuries in other parts of the body. (a) Distribution of concomitant injuries in other parts of the body. (b) Impact of presence and absence of concomitant injuries in other parts of the body on duration of admission.

Of all 1828 patients with maxillofacial fractures, 673 (36.82%) had concomitant injuries in other parts of the body (Fig 6). The three most common injuries were limb injury (327 patients, 17.89%), craniocerebral injury (289 patients, 15.81%), and chest and back injury (273 patients, 14.94%). These patients had usually received systemic treatment in other hospitals and were then transferred to our hospital for facial treatment by a specialist because it would have been impossible to treat the maxillofacial fractures upon arrival at the first hospital. In this study, 220 (32.69%) patients with concomitant injuries in other parts of the body did not receive maxillofacial treatment until 3 weeks after the fracture, whereas only 6.93% of patients without systemic injury received maxillofacial treatment more than 3 weeks after the fracture. This treatment time was actually slightly late and prone to result in the development of an old fracture. Regardless, the 220 patients accounted for 12.03% of the 1828 patients, which is still a relatively high proportion. Therefore, there was a large number of patients with old fractures among all patients with maxillofacial fractures.

We analyzed the causes of injury for patients with concomitant injuries in other body parts. Of these 673 patients, 288 (42.79%) were injured by traffic accidents, 191 (28.38%) by falls from a height, 117 (17.38%) by falls, 65 (9.66%) by assaults, 6 (0.89%) by firearm explosions, 4 (0.59%) incision and puncture wounds, and 2 (0.30%) by animal attacks. Overall, traffic accidents (42.79%) were the major cause of injury in patients who had concomitant injuries in other parts of the body and falls (51.52%) were the most common cause in patients without systemic concomitant injuries.

Postoperative infection analysis

Among the 1558 patients who underwent ORIF of jawbone fractures, 27 developed a postoperative infection. Among these 27 patients, 16 showed symptoms of infection both before and after the surgery, and another 11 had no preoperative infection but developed a postoperative infection, including 8 patients who developed infection before postoperative discharge and three whose infection occurred within 1 month after discharge. Of the 27 patients with postoperative infection, four had an infection in the maxilla, one in the zygoma/zygomatic arch, 21 in the mandible (including 2 in the condyle), and one in the mandible (this patient underwent iliac bone grafting during the surgery).

Overall, the infection after ORIF of jawbone fractures occurred mainly in the mandible (77.78%) and less often in other sites. Infection in the condyle may be related to the absence of negative-pressure drainage, the deep surgical site at this location, or the inability of the postoperatively accumulated blood to drain through the rubber strip. Among patients with postoperative infection of mandibular fractures, the site with the highest incidence of infection was the symphysial site (9 patients), accounting for 42.86% of all parts of the mandible. 

## Discussion

We also selected several reports of maxillofacial trauma involving more than 1000 cases published by Chinese scholars [15-26] to make a preliminary comparison with the present study. Because of limited space, many similar reports with large amounts of data cannot be presented; therefore, only a small part of the data is presented here (Table 2).

**Table 2:** More than 1000 cases of maxillofacial trauma reported by domestic scholars *Total number of cases: The cases without parentheses refer to patients with fractures only. The cases inside parentheses refer to patients with both soft tissue trauma and fractures, and the number within parentheses is the number of patients with fractures. NS, not specified. a: Some of the data in the table are derived from unpublished materials: ① Study 2 (Degree thesis: Li D. The retrospective study of 5887 patients with maxillofacial injuries in Hunan Province; 2010); ② Study 4 (Degree thesis: Caixia L. A retrospective study of 3217 maxillofacial trauma patients; 2021); ③ Study 7 (Degree thesis: Kai C. A retrospective study of 1536 patients with oral and maxillofacial trauma in Qingdao area; 2016); ④ Study 10 (Degree thesis: Qianli Q. Retrospective clinical analysis of 1321 cases of oral and maxillofacial trauma; 2015); ⑤ Study 12 (Degree thesis: Xincai Z. A Retrospective study of 1246 patients of maxillofacial fractures; 2004); ⑥ Study 14 (Degree thesis: Fanglu Z. Retrospective study of 1115 patients with maxillofacial fracture in Kashgar of Xinjiang Uygur Autonomous Region; 2019); ⑦ Study 19 (Degree thesis: Xiao T. The retrospective study of 1022 patients with maxillofacial fractures; 2015); ⑧ Study 21 (Degree thesis: Hui C. A clinical analysis of 1001 cases with oral and maxillofacial trauma; 2012).

Num^a^	First Author	Hospitals Location	Study Time	*Total number of cases	Male-to-Female Ratio	Age of Peak Onset	Cause of Injury	Site of Fracture	Associated injury
1	This study	Chengdu	2018-2021	(1828)	2.48:1	21-30 (24.2%)	Falls (38.95%)	Mandible (37.56%)	Limbs (26.46%)
2	Li D	Changsha + Zhuzhou + Yueyang + Shaoyang + Chenzhou	1990-2009	5887 (4151)	3.78:1	30-39 (23.51%)	Traffic accidents (55.82%)	Mandible (40.32%)	Craniocerebral (30.02%)
3	Yisong L. [15]	Chengdu	1955-2001	3958 (3164)	4.27:1	21-30	Traffic accidents (30.6%)	Mandible (48.4%）	Craniocerebral brain (42.9%)
4	Caixia L.	Hohhot + Baotou + Hulunbuir + Chifeng	2010-2020	3217 (2082)	4.05:1	41-50 (22.4%)	Traffic accidents (45.40%)	Mandible (34.5%)	NS
5	Ke W [16]	Foshan	2003-2009	3050 (2632)	3.9:1	NS	Traffic accidents (34.6%)	Zygoma/zygomatic arch (26.7%)	Craniocerebral (24.6%)
6	Wusiman P [17]	Urumqi + Northern Xinjiang + Southern Xinjiang	2012-2016	2492 (1666)	3.88:1	21-30 (28.3%)	Traffic accidents (41.8%)	Mandible (31.97%)	Limbs (27.5%)
7	Kai C	Qingdao	2002-2015	1536 (1188)	3.95:1	21-30 (30.47%)	Traffic accident (37.17%)	Mandible (42.06%)	Craniocerebral (40.05%)
8	Jun S [18]	Shanghai	2005-2006	1420 (1360)	3.24:1	21-30 (36.22%)	Traffic accidents (45.07%)	Zygoma/zygomatic arch (43.68%)	Limbs (34.26%)
9	Mijiti A [19]	Urumqi	2006-2010	(1350)	4.9:1	21-30 (32.7%)	Traffic accident (49.49%)	Mandible (33.0%)	Craniocerebral (37.0%)
10	Qianli Q	Yantai	2005-2014	1321 (1018)	2.81:1	20-39 (51.93%)	Traffic accident (40.50%)	Jawbone (58.74%)	Craniocerebral (29.21%)
11	Yunfeng Z [20]	Wuhan	2012-2017	(1264)	2.93:1	21-30 (24.13%)	Falls (76.35%)	Mandible (51.61%)	Limbs (37.62%)
12	Xincai Z	Wuhan	1983-2002	(1246)	4.30:1	21-30 (35.96%)	Traffic accident (34.99%)	Mandible (69.93%)	NS
13	Haihua Z [21]	Wuhan	2000-2009	(1131)	3.52:1	19-29 (30.7%)	Traffic accident (52.6%)	Mandible (76.8%)	Eyes (18.5%)
14	Fanglu Z	Kashgar	2011- 2016	(1115)	4.72:1	21-30 (32.74%)	Falls from a height (38.57%)	Mandible (30.26%)	Eyes (35.55%)
15	Wei Z [22]	Changchun	1993-2003	(1097)	4.38:1	21-40 (62.86%)	Traffic accident (57.52%)	Mandible (54.71%)	Craniocerebral (35.10%)
16	Lidong Z [23]	Beijing	1990-2002	(1084)	3.23:1	20-40 (63.5%)	Traffic accident (49.2%)	Mandible (68.9%)	Craniocerebral (10.7%)
17	Min W [24]	Yinchuan	2011-2017	(1081)	4.43:1	20-29 (27.8%)	NS	Mandible (31.90%)	Craniocerebral (38.5%)
18	Ying L [25]	Nanchong	2002-2013	1071 (534)	3.37:1	21-40 (52.75%)	Traffic accident (49.58%)	Mandible (56.00%)	Eyes (11.48%)
19	Xiao T	Changchun	2009-2014	(1022)	3.69:1	20-29 (24.07%)	Traffic accident (37.97%)	Mandible (37.77%)	Limbs (13.80%)
20	Chen C [26]	Beijing	2008-2013	(1009)	2.94:1	20-29 (33.5%)	Traffic accident (42.0%)	Mandible (53.1%)	Limbs (10.57%)
21	Hui C	Xi'an	2008-2011	1001 (952)	2.80:1	20-29 (30.07%)	Traffic accidents (48.45%)	NS	Eyes (17.08%)

Different reports provide different interpretations of facial trauma caused by traffic accidents. We investigated the definition of a traffic accident and found that one of the core elements is that a vehicle must be involved, and it may be either a motor vehicle or a non-motor vehicle. Therefore, the scope of the definition is very broad. However, the severity and clinical characteristics of the trauma caused by bicycle accidents are very different from that caused by traffic accidents. In the present study, therefore, we only included patients with injuries caused by motor vehicle accidents; we did not consider injuries caused by traffic accidents involving non-motor vehicles. Some studies even consider motorcycles and cars as two different causes in statistics [27]. If a bicycle does not collide with a car, the accident will likely be classified as a fall.

Broadly speaking, fall injuries include falls from a height and walking/cycling falls. Although the term "fall" is used in both types of injuries, the causes and severity of the injury are different. Therefore, the studies were conducted separately for the two injuries.

In this study, the proportion of facial fractures caused by falls from a height reached 16.90%, which was only secondary to falls and traffic accidents. In another study, the proportion of falls from a height reached 38.57%, ranking first among all causes (Degree thesis: Fanglu Z, 2019). We reviewed the patient's medical history and found that the specific causes of falls from a height were accidents when adults were working at a height, accidents when minors were fighting at home or school, and falling off a mountain or accidentally falling from a high place in daily life. These patients' injuries are generally more serious and often occur simultaneously with multiple injuries throughout the body. To reduce the incidence of falls from a height, it is necessary to provide complete protective measures and supervision systems for people at high risk and strengthen the safety education of minors so that they can identify risks in the environment.

The patients in the present study were mainly from Sichuan and comprised more male than female patients. The male: female ratio was 2.48:1, which was lower than that in the study by Yisong et al. [15] (4.27:1) and Ying et al. [25] in northern Sichuan (3.37:1). Males are more involved than females in high-intensity, high-risk social activities, which explains why males are more susceptible to maxillofacial trauma. In recent years, however, females have also become increasingly more involved in these activities, which may be one reason why our hospital's male: female ratio of patients with maxillofacial fractures has decreased in recent years.

In this study, maxillofacial fractures caused by traffic accidents accounted for 28.12% of the total, much lower than the described in the national college textbook Oral and Maxillofacial Surgery [28]. In fact, all of the studies listed in Table 2 showed a rate of <60% of traffic accident injuries. In addition, China's implementation of the "drunk driving sentence" policy, the strengthening of penalties in 2011, and the constant increase in people's safety awareness have reduced the probability of traffic accidents.

The 21 to 50-year-old age group had the highest incidence of maxillofacial fractures in this study, accounting for 61.54%. This result is close to the 19 to 44-year age group (65.6%) in the study by Al-Bokhamseen et al. [29]. This is because 21 to 50-year-olds are the main labor force of society, frequently participate in various social activities, and are more exposed to various risk factors. Thus, they are more likely to develop maxillofacial fractures.

In this study, pediatric patients had an average of 2.09 fractures per person, lower than adult patients (3.15 sites). And among pediatric patients, the proportion of midface fractures (31.61%) has significantly decreased. The low mineralization of bone may explain these differences, the large quantities of facial soft tissues, the presence of unerupted teeth, greater cranium-to-face ratio, the lack of paranasal sinus pneumatization, and being protected by parents and schools [30-34].

In this study, mandibular fractures were the most common type of fracture, consistent with the findings of Soundarya et al. [35]. and Adesina et al. [36]. This may be related to the protruding nature of the mandible and its arch shape in the facial position [37]. The symphysial (682 cases, 36.16%) and condyle (656 cases, 34.78%) are also weak sites of the mandible and prone to fractures. When falling forward, the chin and mandibular body are likely to hit the ground first; simultaneously, because the stress is transferred backward and upward, the condyle is also subject to strong shocks and is prone to fracture [33,38]. Therefore, patients with symphysial or submandibular trauma should be evaluated by a specialist to reduce the risk of a missed diagnosis.

We also found that the mandible is more susceptible to postoperative infection than other maxillofacial structures, which is consistent with the findings of a study by Liping (Degree thesis: Liping G, 2020) on risk factors for infection after maxillofacial fractures. This may be partly due to the easy entry of saliva into the surgical area through an intraoral incision, a tooth extraction socket, or a gum laceration, exposing the fracture site and fixation material to bacteria [39] and resulting in infection. Another cause may be trauma or intraoral penetration through the mouth due to intraoperative procedures. To prevent postoperative infection, it is necessary to scientifically design the surgical incision before surgery, standardize the operation procedures during the operation, strictly suture the wound in the mouth, and rationally apply drainage. After surgery, it is necessary to strengthen the oral hygiene maintenance of the operative area and antibacterial drugs should be applied if necessary. An important issue often ignored is that postoperative eating, rinsing, and gargling must be done gently to ensure that the soft tissue wounds can heal and that infection does not develop. Of course, an infection may also be related to the doctor's surgical procedures. Each of our surgeons has undergone strict and standardized training, paying great attention to aseptic technique, but we still need to conduct separate studies on postoperative infection cases in order to find ways to reduce infection rates.

In this study, limb injury (17.89%), skull and brain injury (15.81%), and chest and back injury (14.94%) were the three most common concomitant bodily injuries. In a study by Mahat et al. [40], skull and brain injury (23%) was the most common concurrent injury. By Fanglu (Degree thesis: Fanglu Z, 2019), the most common concurrent injury was eye injury (35.55%), indicating that the concomitant bodily injuries in patients with maxillofacial fractures are various. Generally, however, most reports have shown that skull and brain injury is the most common concomitant injury. When a maxillofacial surgeon provides care to a trauma patient, he or she should be alert to abnormalities in other parts of the patient's body and give priority to the treatment of injuries that endanger the patient's life. Multispecialty collaboration may be needed in such cases. For patients with craniocerebral injury, symptoms such as intracranial hemorrhage and hypertension should also be detected on time to avoid a missed diagnosis. In addition, the maxillofacial surgery department in many cities in China is located in stomatological hospitals rather than general hospitals, which often makes it impossible for maxillofacial surgeons to cooperate with doctors of other specialties to perform maxillofacial surgery along with surgeries involving other parts of the body. This will theoretically delay the timing of facial treatments and increase the number of old maxillofacial fractures.

Regarding the choice of treatment methods, if a maxillofacial fracture is not severely displaced and the impact on facial morphology and stomatognathic functions is not obvious, then the surgical effect will be limited, and conservative treatment can reduce soft tissue dissection, neurovascular injury, scar formation, and incision pain [41]. For these patients, conservative treatment is a more appropriate choice.

## Conclusions

In summary, maxillofacial fractures tend to occur in male patients and, more often, in young and middle-aged people. The main causes of injury are falls and traffic accidents, and these fractures are often concomitant with limb injuries, skull and brain injuries, and chest and back injuries. In patients with jawbone fractures, multiple fractures are more common than a single fracture, and the mandible is most prone to fractures. ORIF is currently the main treatment method for maxillofacial fractures, and conservative treatment may also be considered depending on the situation. The development of surgical tools and digital technologies has greatly improved the diagnosis, surgical planning, and treatment effect of maxillofacial fractures.
